# Psychological Status and its Influencing Factors of Staff in a District of Shenzhen: A Retrospective Study

**DOI:** 10.62641/aep.v53i1.1699

**Published:** 2025-01-05

**Authors:** Junli Zheng, Meilian Jiang, Kai Zheng, Jianfeng Li, Ling Ye, Jiaqi Wu, Jun Feng, Xiaoting Luo, Yanxia Liao, Zhicong Chen

**Affiliations:** ^1^Department of Chronic Disease, Longhua District Center for Chronic Disease Control/Mental Health, 510080 Shenzhen, Guangdong, China; ^2^Public Health Service Center, Bao’an District, 518100 Shenzhen, Guangdong, China

**Keywords:** Self-rating Anxiety Scale, Self-rating Depression Scale, Pittsburgh Sleep Quality Index

## Abstract

**Background:**

Anxiety, depression, and sleep disorders, as psychological and emotional diseases, have serious impact on people's physical and mental health, and receive increasing academic attention. This study aimed to examine anxiety, depression, and sleep disorder of staff in a district of Shenzhen and to provide the basis for the development of targeted intervention measures to improve the psychological status of cadres.

**Methods:**

Based on the psychological evaluation data of staff cadres in a district of Shenzhen City obtained from January to December 2020, a stratified sampling method was adopted to randomly select two streets and three communities in each street. A total of six communities were selected as investigation units. All participants filled out the Self-rating Anxiety Scale (SAS), Self-rating Depression Scale (SDS), and Pittsburgh Sleep Quality Index (PSQI). Chi-square test and multiple logistic regression analysis were performed using R4.2.0 statistical software.

**Results:**

A total of 705 effective psychological assessment questionnaires were matched, and there were 71 (10.13%) positive results on SAS, 156 (22.13%) positive results on SDS, and 264 (37.45%) positive results on PSQI. Chi-square test results showed that the detection rates of anxiety and depression were significantly different among the staff cadres of different genders and different educational levels (*p* < 0.05). The detection rate of sleep disorder of government officials significantly differed among different age groups (*p* < 0.05). The logistic regression analysis showed that the detection rates of anxiety, depression, and sleep disorder of female cadres and workers were significantly higher than those of male cadres and workers (*p* < 0.05). The detection rates of anxiety and depression of the staff with bachelor's degree and graduate degree were significantly lower than those of the staff with a college degree or below (*p* < 0.05).

**Conclusion:**

The detection rates of anxiety and depression are different among staff of different genders and different education levels in a district of Shenzhen, where female staff and those with lower education levels have higher detection rates.

## Introduction

With the continuous development of social science, technology and medicine, the medical level has greatly improved. However, the variety and number of diseases suffered by the population have also increased significantly. In particular, psychological and emotional diseases such as anxiety, depression, and sleep disorders have received widespread attention in recent years. Seasonal affective disorder (SAD) is a chronic anxiety disorder with a low self-healing effect. If left untreated, it may affect multiple areas of function, including education, employment, personal development, and relationships [1]. Depression is a mood disorder that causes persistent feelings of sadness and loss [2]. Sleep disturbance is another common health complaint and affects about 10%–25% of the general population [3]. According to the World Health Organization statistics, depressive disorder and anxiety disorder are among the most prevalent mental disorders globally, affecting approximately 5%–6% of people worldwide and with lifetime prevalence rates as high as 30%, respectively [4]. Long-term negative emotions can affect people’s feelings, thinking, and behavior, leading to a variety of mental illnesses and physical problems. Mental illness caused by negative emotions is not a sign of personal weakness or a state that can be wished away, and it tends to be intermittent, with episodes lasting for weeks or months. While symptoms tend to spontaneously resolve over time [5], some form of treatment is important to reduce the likelihood of recurrence.

Staff are important forces for social development, and their physical and mental health is of great significance for their work performance and individual development. Anxiety, depression, and sleep disorder are common psychological problems of cadres and workers, which can have negative effects on individuals and society. Understanding the research progress, influencing factors, and prevention measures of anxiety, depression, and sleep disorders of cadres and workers is of paramount importance for formulating effective intervention strategies and maintaining the physical and mental health of cadres and workers. Therefore, by analyzing the symptoms and risk factors of anxiety, depression, and sleep disorders of cadres in a district of Shenzhen, we aimed to provide a basis for formulating targeted intervention measures to improve the psychological status of cadres.

## Materials and Methods

### Research Subjects

The psychological assessment data of 705 staff in a district of Shenzhen from January 1, 2020, to December 31, 2020, were retrospectively analyzed. Inclusion criteria were as follows: ① staff, employees, and temporary employees who are on duty; ② age 18–60 years; ③ complete information available. Exclusion criteria were as follows: ① incomplete information; ② fill in the duplicate ID; ③ logic conflicts existing before and after questionnaire. The questionnaires were distributed online and filled in through the psychological assessment system of the Center. The questionnaire consisted of four parts as follows: ① basic information; ② Self-rating Anxiety Scale (SAS) [6], where the SAS standard score ≥50 indicated the presence of anxiety symptoms, while that below 50 indicated no anxiety symptoms; ③ Self-rating Depression Scale (SDS) [6], where the SDS standard score ≥50 indicated the presence of depressive symptoms, while that below 50 indicated no depressive symptoms; ④ Pittsburgh Sleep Quality Index (PSQI) [7], where according to clinical studies, the score of 8 points or more indicated the presence of sleep disorders, while that below 8 points indicated no sleep disorder. All data were approved by the Ethics Review Committee on Biomedical Research of the Shenzhen Longhua District Center for Chronic Disease Control (mental health center) before use (ethics approval number: 20240308001). All data were collected with the informed consent of each participant.

### Questionnaires

The Zung SAS is a psychological assessment tool designed to quantify a person’s level of anxiety [6]. Developed by Zung *et al*. [6] in 1990, the scale aims to measure the severity of anxiety symptoms in individuals.

The Zung SDS is an instrument used for measuring the presence and severity of depressive symptoms in individuals [6]. Developed by Zung *et al*. [6], this scale has been widely employed in various settings to assess depression and its associated factors across diverse populations.

The PSQI, developed by Buysse *et al*. [7], is a self-report assessment tool that evaluates sleep quality over a one-month period. A global score and seven component scores can be derived from the scale [7, 8]. The component scores are the following: subjective sleep quality, sleep latency, sleep duration, sleep efficiency, sleep disturbances, use of sleeping medications, and daytime dysfunction [7, 8]. Each component is scored on a scale from 0 to 3, with the total score ranging from 0 to 21, where a higher score describes poorer sleep quality [7, 8].

### Research Method

#### Sampling Method

In this study, two streets in a district of Shenzhen were randomly selected, and three communities were selected from each street. A total of six communities were selected as research units. A total of 713 questionnaires were obtained, of which 705 were valid, with an effective rate of 98.88%.

#### Survey Methods

After consulting relevant literature, a questionnaire was designed in line with the purpose of the investigation. The contents of the questionnaire included personal basic information, educational level, working years, marital status, SAS, SDS, and PSQI scales, and the questionnaire was collected through an online questionnaire survey.

### Statistical Analysis

The questionnaire was exported to MS Excel to establish a database, and the data were summarized, cleaned, and analyzed with R 4.2.0 (Beijing Foreign Studies University, Beijing, China) statistical software. Statistical data rates were described, and the chi-square test was used for comparison between groups. The factors affecting anxiety, depression, and sleep disorders were analyzed by multivariate logistic regression analysis, and the assigned variables are shown in Table 1. Bilateral tests were adopted, with an alpha level of 0.05. *p* values of 0.05 or lower were considered statistically significant.

**Table 1.** **Assigned variables of multivariate logistic regression analysis**.

Variable	Assigned values
Depression	0, normal; 1, depression
Anxiety	0, normal; 1, anxiety
Sleep disorder	0, normal; 1, sleep disorder
Age	1, 18–30 years; 2, 30–40 years; 3, 40–50 years; 4, ≥50 years
Sex	1, male; 2, female
Education level	1, college and below; 2, undergraduate; 3, master’s degree or above
Marital status	1, unmarried; 2, married; 3, other
Work	1, 0–4 years; 2, 5–14 years; 3, 15–24 years; 4, ≥25 years

## Results

### Population Characteristics

There were 229 males (32.48%) and 476 females (67.52%) in the study population. A total of 42 individuals had a college degree or below (5.96%), 494 individuals had a bachelor’s degree (70.07%), and 169 individuals had a master’s degree (23.97%). There were 71 (10.13%) positive results on SAS, 156 (22.13%) positive results on SDS, and 264 (37.45%) positive results on PSQI. A total of 428 individuals (60.71%) were married (Table 2).

**Table 2.** **Characteristics of staff in a district of Shenzhen (n = 705)**.

Item		Number of cases	Percent
Sex			
	Male	229	32.48%
	Female	476	67.52%
Age (years)			
	18–30	186	26.38%
	30–40	318	45.11%
	40–49	141	20.00%
	≥50	60	8.51%
Level of education			
	College and below	42	5.96%
	Undergraduate	494	70.07%
	Master’s degree or above	169	23.97%
PSQI score			
	<8	441	62.55%
	≥8	264	37.45%
Marital status			
	Unmarried	250	35.46%
	Married	428	60.71%
	Other	27	3.83%
SAS score			
	<50	634	89.87%
	≥50	71	10.13%
SDS score			
	<50	549	77.87%
	≥50	156	22.13%
Work (years)			
	<5	222	31.49%
	5–14	278	39.43%
	15–24	139	19.72%
	≥25	66	9.36%

PSQI, Pittsburgh Sleep Quality Index; SAS, Self-rating Anxiety Scale; SDS, Self-rating Depression Scale.

### Detection Rates of Anxiety, Depression, and Sleep Disorder among Staff

There were significant differences in the detection rates of anxiety (χ^2^ = 4.64, *p* = 0.031; χ^2^ = 7.02, *p* = 0.008) and depression (χ^2^ = 7.90, *p* = 0.019; χ^2^= 8.84, *p* = 0.012) among workers and cadres of different genders and different education levels. The detection rate of sleep disorder significantly differed among government officials of different ages (χ^2^ = 8.05, *p* = 0.045). There were no significant differences in the detection rate of sleep disorder among cadres and employees with different marital statuses and different working years (*p* > 0.05) (Table 3).

**Table 3.** **Presence of anxiety, depression, and sleep disorder in staff stratified by various variables [cases (%)]**.

Variable		SAS		χ ^2^	*p*	SDS		χ ^2^	*p*	PSQI		χ ^2^	*p*
		<50 (n = 634)	≥50 (n = 71)			<50 (n = 549)	≥50 (n = 156)			<8 (n = 441)	≥8 (n = 264)		
Sex				4.64	0.031			7.02	0.008			3.19	0.074
	Male	214 (33.75)	15 (21.13)			192 (34.97)	37 (23.72)			154 (34.92)	75 (28.41)		
	Female	420 (66.25)	56 (78.87)			357 (65.03)	119 (76.28)			287 (65.08)	189 (71.59)		
Age (years)				2.96	0.397			1.47	0.690			8.05	0.045
	18–30	169 (26.66)	17 (23.94)			140 (25.50)	46 (29.49)			132 (29.93)	54 (20.45)		
	30–40	290 (45.74)	28 (39.44)			248 (45.17)	70 (44.87)			191 (43.31)	127 (48.11)		
	40–50	124 (19.56)	17 (23.94)			114 (20.77)	27 (17.31)			81 (18.37)	60 (22.73)		
	≥50	51 (8.04)	9 (12.68)			47 (8.56)	13 (8.33)			37 (8.39)	23 (8.71)		
Level of education				7.90	0.019			8.84	0.012			3.13	0.209
	College and below	33 (5.21)	9 (12.68)			25 (4.55)	17 (10.90)			21 (4.76)	21 (7.95)		
	Undergraduate	452 (71.29)	42 (59.15)			392 (71.40)	102 (65.38)			311 (70.52)	183 (69.32)		
	Master’s degree or above	149 (23.50)	20 (28.17)			132 (24.04)	37 (23.72)			109 (24.72)	60 (22.73)		
Marital status				4.47	0.215			2.45	0.294			2.20	0.332
	Unmarried	228 (35.96)	22 (30.99)			190 (34.61)	60 (38.46)			160 (36.28)	90 (34.09)		
	Married	380 (59.94)	48 (67.61)			335 (61.02)	93 (59.62)			261 (59.18)	167 (63.26)		
	Other	26 (4.10)	1 (1.41)			24 (4.37)	3 (1.92)			20 (4.54)	7 (2.65)		
Work (years)				4.47	0.215			4.06	0.255			7.40	0.060
	<5	197 (31.07)	25 (35.21)			163 (29.69)	59 (37.82)			154 (34.92)	68 (25.76)		
	5–14	258 (40.69)	20 (28.17)			224 (40.80)	54 (34.62)			168 (38.10)	110 (41.67)		
	15–24	121 (19.09)	18 (25.35)			111 (20.22)	28 (17.95)			78 (17.69)	61 (23.11)		
	≥25	58 (9.15)	8 (11.27)			51 (9.29)	15 (9.62)			41 (9.30)	25 (9.47)		

### Multivariate Logistic Regression Analysis of Factors Influencing Anxiety and Depression of Staff

We further carried out multivariate logistic regression analysis with anxiety and depression as the dependent variables, and gender and education level as the independent variables. The results showed that, with male as reference, the detection rates of anxiety (odds ratio [OR] = 2.23, 95% confidence interval [CI]: 1.19–4.17, *p* = 0.012) and depression (OR = 1.88, 95% CI: 1.22–2.88, *p* = 0.004) were significantly higher in females. Taking college education or below as reference, more highly educated individuals had significantly lower detection rates of anxiety (OR = 0.32; 95% CI: 0.14–0.74, *p* = 0.008 and OR = 0.37, 95% CI: 0.14–0.98, *p* = 0.046) and depression (OR = 0.34, 95% CI: 0.17–0.66, *p* = 0.002 and OR = 0.27, 95% CI: 0.12–0.60, *p* = 0.001) (Table 4).

**Table 4.** **Multivariate logistic regression analysis of anxiety and depression in staff**.

Variable		SAS					SDS				
		β	S.E	Z	*p*	OR (95% CI)	β	S.E	Z	*p*	OR (95% CI)
Sex											
	Male					1.00 (Reference)					1.00 (Reference)
	Female	0.80	0.32	2.51	0.012	2.23 (1.19–4.17)	0.63	0.22	2.88	0.004	1.88 (1.22–2.88)
Education level											
	College and below					1.00 (Reference)					1.00 (Reference)
	Undergraduate	−1.14	0.43	−2.66	0.008	0.32 (0.14–0.74)	−1.09	0.35	−3.14	0.002	0.34 (0.17–0.66)
	Master’s degree or above	−0.99	0.50	−1.99	0.046	0.37 (0.14–0.98)	−1.30	0.40	−3.24	0.001	0.27 (0.12–0.60)

OR, odds ratio; CI, confidence interval.

## Discussion

Sleep quality is a comprehensive indicator that encompasses sleep duration, sleep depth, sleep efficiency, and other aspects. Poor sleep quality seriously affects the health of the human body, and different degrees of anxiety, depression, and sleep disorders can lead to psychological stress and symptoms of headache. It can also lead to mental illness, cardiovascular disease, and intestinal disease, and even increase the probability of accidents and deaths [9, 10]. This study showed that the detection rates of anxiety, depression, and sleep disorder of female cadres and workers were significantly higher than those of male cadres and workers, which may be related to female menopause. Previous studies have shown that sleep quality, depression, menopausal symptoms, and quality of life of menopausal women are significantly correlated [11, 12, 13]. Among adolescents, the incidence of depression in females is significantly higher than that in males [14, 15, 16], which is consistent with the results of this study. The results of this study showed that the detection rates of anxiety and depression of staff with a low education level were significantly higher than those of staff with a high education level, which may be related to the psychological status of staff with a high education level and better knowledge of how to express emotions. Chrzastek *et al*. [17] conducted a study on 1975 elderly outpatients and showed that the incidence of depressive symptoms in the elderly with a low education level was significantly higher than that in the elderly with a high education level. Figueiredo-Braga *et al*. [18] showed that adolescent age and education level had a protective effect on depression in patients with systemic lupus erythematosus.

Depression can affect people’s behavior, life, and work, causing physical and mental damage, and in severe cases it can even lead to suicide [19, 20]. Previous studies have shown that the severity of depression correlates with suicide risk [21, 22]. According to Michaelides and Zis [23], the link between mood disorders and acute pain is increasingly significant since the link is bidirectional, and both act as risk factors for each other. Götze *et al*. [24] have shown that among cancer patients, working-age survivors are more likely to suffer from anxiety and depression than older survivors, and female survivors are more likely to suffer from anxiety and depression than male survivors. Depression and anxiety are the most prevalent mental health problems in the workplace, costing the global economy up to $1 trillion a year [25]. In today’s society, where some diseases occur at a younger age, mastering the psychological condition of the staff and implementing targeted intervention measures for the staff with depression are important measures to alleviate depression, improve work efficiency, and maintain a healthy body.

In recent years, driven by policies promoting innovation, development, and reform, the comprehensive quality requirements for staff have steadily risen. The pressure faced by staff has gradually increased due to evolving societal demands and heightened work expectations. Consequently, emotional issues among staff stemming from various pressures have become more pronounced. Relevant authorities should prioritize addressing the emotional well-being of staff. Proactive measures should be taken to establish psychological counseling facilities and implement mental health education programs to enhance the mental health of staff.

## Conclusion

The detection rates of anxiety and depression are different among staff of different genders and different education levels in a district of Shenzhen, where female staff and those with lower education levels show higher detection rates.

## Availability of Data and Materials

The datasets used and/or analysed during the current study were available from the corresponding author on rea-sonable request.
